# Combined Effect of Low-Density Lipoprotein Cholesterol and Homocysteine on Major Adverse Cardiovascular Events in Coronary Heart Disease: A Retrospective Cohort Study

**DOI:** 10.31083/RCM46290

**Published:** 2026-03-16

**Authors:** Baozhen Zhu, Xingyu Luo, Peng Wu, Yuru Ma, Bo Wu, Ru Yan, Tianshui Ma, Jiawei Yang, Ziyi Wang, Guangzhi Cong, Shaobin Jia

**Affiliations:** ^1^The First Clinical College, Ningxia Medical University, 750004 Yinchuan, Ningxia, China; ^2^Department of Intervention, Tongxin County People’s Hospital, 751300 Wuzhong, Ningxia, China; ^3^Department of Cardiology, Peking University First Hospital, 100034 Beijing, China; ^4^Heart Centre & Department of Cardiovascular Diseases, General Hospital of Ningxia Medical University, 750004 Yinchuan, Ningxia, China; ^5^The First School of Clinical Medicine, Southern Medical University, 51000 Guangzhou, Guangdong, China; ^6^Institute of Medical Sciences, General Hospital of Ningxia Medical University, 750004 Yinchuan, Ningxia, China; ^7^National Health Commission Key Laboratory of Metabolic Cardiovascular Diseases Research, Ningxia Medical University, 750004 Yinchuan, Ningxia, China; ^8^Ningxia Key Laboratory of Vascular Injury and Repair Research, Ningxia Medical University, 750004 Yinchuan, Ningxia, China

**Keywords:** coronary heart disease, low-density lipoprotein cholesterol, homocysteine, major adverse cardiovascular events, combined effect

## Abstract

**Background:**

Residual cardiovascular risk remains substantial despite aggressive low-density lipoprotein cholesterol (LDL-C) lowering in coronary heart disease (CHD). Consequently, this elevated risk has spurred the search for non-lipid targets, such as homocysteine (HCY). However, the combined effect of HCY with LDL-C and the overall potential for combined risk stratification remain unclear.

**Methods:**

This retrospective cohort study included patients with CHD confirmed by coronary angiography or computed tomography angiography at the General Hospital of Ningxia Medical University between January 2019 and December 2021. Participants were stratified by baseline LDL-C levels (<1.8 vs. ≥1.8 mmol/L) and HCY (<15 vs. ≥15 μmol/L). Major adverse cardiovascular events (MACEs) were employed as the primary endpoint, defined as a composite of all-cause death, stroke, non-fatal myocardial infarction, or unplanned revascularization.

**Results:**

A total of 744 MACEs were recorded during the 25-month follow-up. Elevated levels of LDL-C (adjusted hazard ratio (aHR) = 1.38, 95% confidence interval (CI): 1.09–1.73) and HCY (aHR = 1.47, 95% CI: 1.19–1.81) were independently associated with a higher risk of MACEs. The risk was synergistic when both factors were elevated, as patients in the high LDL-C and high HCY group had a significantly increased risk (aHR = 1.97, 95% CI: 1.34–2.90) compared to the reference group with low levels.

**Conclusion:**

LDL-C and HCY are independent predictors of MACEs in patients with CHD, and the combined use of these indices improves risk stratification. Thus, integrating these indices into clinical practice could improve personalized management strategies and outcomes in this high-risk population.

## 1. Introduction

Cardiovascular disease (CVD) persists as a predominant cause of global mortality [1]. Within this category, coronary heart disease (CHD) represents a significant component, often resulting in serious outcomes including death, myocardial infarction, and the need for revascularization procedures. Low-density lipoprotein cholesterol (LDL-C) is an acknowledged contributor to atherosclerosis and serves as a fundamental biomarker for risk evaluation and management in atherosclerotic cardiovascular disease [2]. Robust evidence confirms a causative link between LDL-C and CHD [3], leading international guidelines to uniformly advocate for aggressive LDL-C reduction in secondary prevention strategies [3, 4]. However, achieving target LDL-C levels does not eradicate all cardiovascular risk, indicating the necessity to discover other modifiable factors.

Conventional risk factors such as hypertension, diabetes, dyslipidemia, and smoking do not completely elucidate this residual risk [5]. Homocysteine (HCY), a sulfur-containing amino acid produced during methionine metabolism, has been recognized as an independent cardiovascular risk factor [6, 7]. Increased HCY concentrations are correlated with impaired endothelial function, enhanced oxidative stress, and a propensity for thrombosis [8, 9, 10]—key processes that facilitate the development and instability of atherosclerotic plaque. Longitudinal research has demonstrated that individuals with high HCY levels face a greater likelihood of CHD and mortality from all causes [11]. However, the causal role of HCY remains debated, and large-scale trials of HCY-lowering with B vitamins have yielded largely neutral results [12], leading to the current treatment that does not recommend such therapy for CHD management.

The pro-atherogenic effects of HCY may intersect with and amplify those of LDL-C. Biologically, HCY-induced endothelial dysfunction promotes LDL retention and modification, while concomitant oxidative stress accelerates foam cell formation and plaque progression [13, 14], suggesting a plausible synergistic risk. Furthermore, the role of lipids exhibits paradoxes; whereas LDL-C unequivocally drives atherosclerosis, its link to arrhythmias like atrial fibrillation is complex, as highlighted in a pertinent meta-analysis [15]. Despite this mechanistic interplay, the combined effect of LDL-C and HCY on clinical outcomes in CHD remains inadequately evaluated, with most prior studies examining these factors in isolation.

Owing to the constraints of relying on a single biomarker, the integration of multiple biomarkers into clinical practice could refine cardiovascular risk prediction. This study therefore sought to examine the individual and combined influences of LDL-C and HCY on the incidence of major adverse cardiovascular events (MACEs) in patient with CHD.

## 2. Materials and Methods

### 2.1 Study Design and Population

We performed a retrospective cohort study involving patients diagnosed with CHD who received coronary angiography or coronary computed tomography angiography at the General Hospital of Ningxia Medical University from January 2019 to December 2021. CHD was characterized by the presence of ≥50% stenosis in one or more major coronary arteries. The institutional ethics review board approved the study protocol and waived the need for individual informed consent due to the retrospective design.

Inclusion criteria were: (1) patients aged 18 years or older; (2) a confirmed diagnosis of CHD (encompassing stable or unstable angina or myocardial infarction); (3) availability of baseline LDL-C and HCY measurements. Exclusion criteria included: (1) absent or incomplete clinical or laboratory data; (2) severe concomitant illnesses (such as advanced cancer or end-stage renal disease); (3) loss to follow-up within the 25-month study period.

**Fig. 1.**
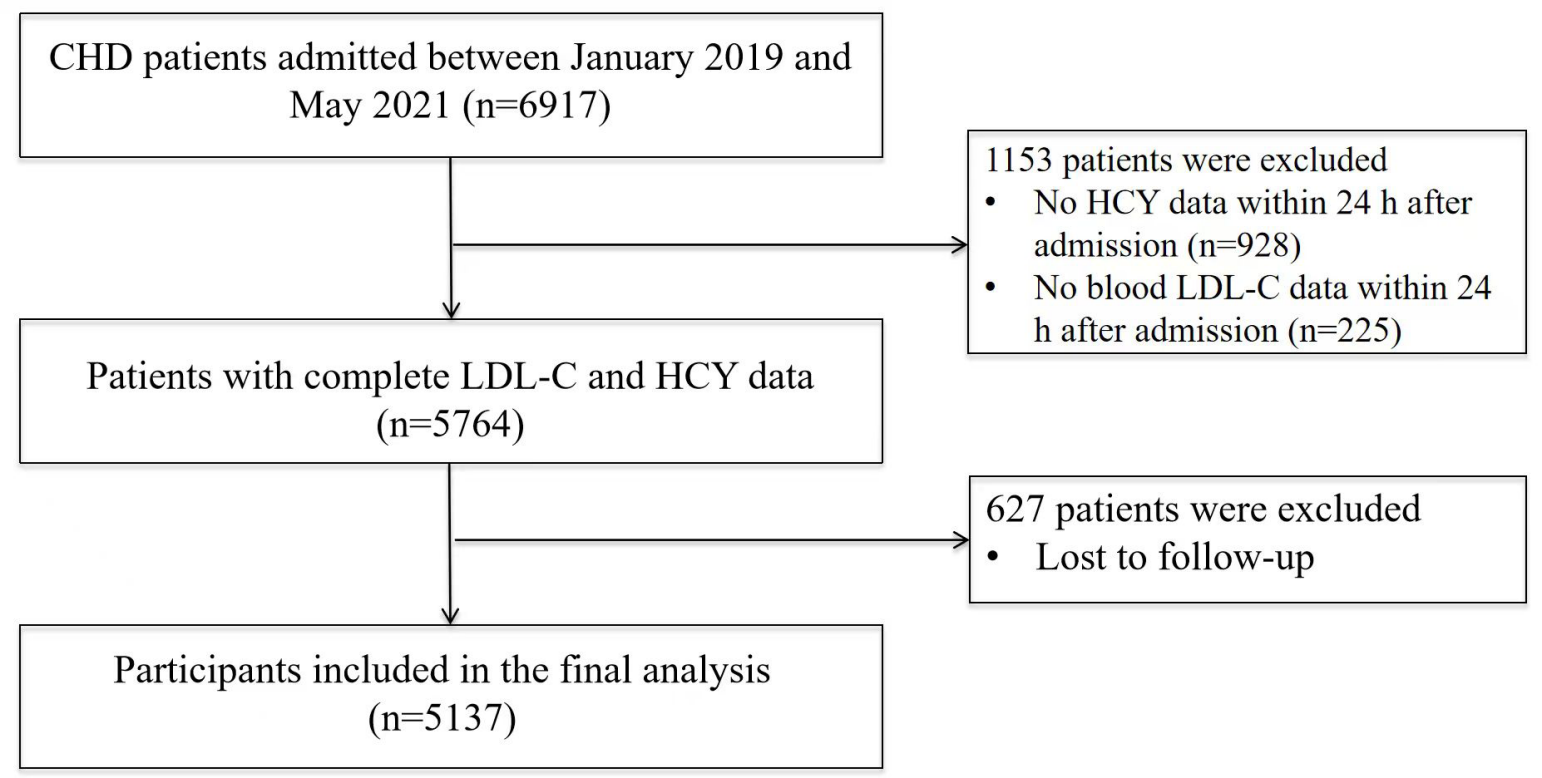
**Flowchart illustrating the selection process of study participants**. CHD, coronary heart disease.

From an initial screening of 6917 patients, 1153 were excluded due to insufficient data, and an additional 627 were lost to follow-up, yielding a final analytical cohort of 5137 individuals (Fig. 1). The study protocol was approved by the General Hospital of Ningxia Medical University ethics committee, with a waiver for patient informed consent.

Participants were stratified by baseline LDL-C and HCY levels using clinically relevant cut-off points. The LDL-C cut-off of 1.8 mmol/L was selected in accordance with the recommended target for secondary prevention in current international guidelines [3, 4]. The HCY cut-off of 15 µmol/L is widely used to define hyperhomocysteinemia in clinical practice and epidemiological studies in our region, and aligns with the reference standard of our institutional laboratory.

### 2.2 Data Collection

Demographic information, lifestyle factors, clinical history (including age, sex, smoking status, alcohol consumption, hypertension, diabetes, and body mass index [BMI]), and laboratory parameters (LDL-C, HCY, total cholesterol [TC], triglycerides [TG], high-density lipoprotein [HDL-C], fasting glucose, creatinine) were obtained from electronic medical records. Blood specimens were drawn from a peripheral vein into Ethylenediaminetetraacetic acid containing tubes within 24 hours of hospital admission and analyzed using a Sysmex automated hematology analyzer (Sysmex Corporation, Kobe, Japan) and a Beckman Coulter series automated biochemistry analyzer (Beckman Coulter, Inc., Brea, CA, USA). Information on interventional procedures, including percutaneous coronary intervention (PCI), was also documented.

### 2.3 Follow-Up and Endpoints

The follow-up period extended to 25 months after patient discharge. The primary endpoint was the occurrence of MACEs, a composite of all-cause mortality, stroke, acute myocardial infarction (AMI), or unplanned revascularization. Follow-up data were acquired through review of electronic health records or structured telephone interviews. Standardized follow-up procedures were implemented by trained staff, and any ambiguous events were reviewed by two senior clinicians.

All-cause mortality was defined as death from any cause. Stroke was identified as a disabling neurological deficit attributable to cerebral ischemia or hemorrhage. Non-fatal myocardial infarction was diagnosed based on ischemic symptoms, elevated cardiac biomarker levels, characteristic electrocardiographic changes, or imaging evidence of new myocardial necrosis, in the absence of fatal outcome. Unplanned revascularization referred to any urgent coronary procedure performed in response to an acute coronary syndrome or other acute ischemic event.

### 2.4 Statistical Analysis

Continuous variables are summarized as mean ± standard deviation (SD) or median [interquartile range (IQR)], and categorical variables as counts and percentages. Group comparisons utilized one-way Analysis of Variance (ANOVA) for normally distributed variables, the Kruskal–Wallis test for non-normally distributed variables, and the chi-square test for categorical variables.

The continuous associations of LDL-C and HCY with MACE risk were visualized using generalized additive models with smoothing splines. Cumulative event incidence was plotted with Kaplan–Meier curves, and group differences were assessed with the log-rank test. Univariate and multivariable Cox proportional hazards models were employed to compute hazard ratios (HRs) and corresponding 95% confidence intervals (CIs). Multivariable models were adjusted for age, sex, hypertension, diabetes, smoking status, alcohol use, TC, TG, BMI, and creatinine levels.

All statistical analyses were conducted using R software (version 4.1.0; R Foundation for Statistical Computing, Vienna, Austria) and EmpowerStats (version 4.0; X&Y Solutions, Inc., Boston, MA, USA), a statistical platform commonly used in biomedical research. A two-sided *p*-value below 0.05 was deemed statistically significant.

## 3. Results

### 3.1 Baseline Characteristics

Table 1 displays the baseline characteristics for the 5137 participants, categorized according to their LDL-C and HCY levels. Significant differences were observed across groups regarding demographic, lifestyle, and clinical variables (all *p* < 0.05 unless specified). Subjects in the high HCY categories (low LDL-C & high HCY; high LDL-C & high HCY) were older (mean age 65.04 ± 10.59, 62.89 ± 11.50 years, respectively) and more frequently male (77.1%, 73.6%, respectively). The highest rate of smoking was found in the high LDL-C & high HCY group (51.3%). The prevalence of hypertension and diabetes also differed significantly among the subgroups. Relevant laboratory values, including fasting glucose, HCY, LDL-C, and creatinine, also exhibited significant variation (all *p* < 0.001).

**Table 1.** **Baseline characteristics of participants stratified by LDL-C and HCY levels**.

Variable		Total (N = 5137)	Low LDL-C & low HCY (n = 448)	High LDL-C & low HCY (n = 1095)	Low LDL-C & high HCY (n = 957)	High LDL-C & high HCY (n = 2637)	*p* value
Demographics							
	Age, years (SD)	62.66 ± 11.11	61.65 ± 10.30	60.42 ± 10.44	65.04 ± 10.59	62.89 ± 11.50	<0.001
	Male, n (%)	3525 (68.6)	252 (56.2)	595 (54.3)	738 (77.1)	1940 (73.6)	<0.001
	BMI, kg/m^2^ (SD)	25.10 ± 3.39	24.69 ± 3.28	25.19 ± 3.30	24.92 ± 3.46	25.21 ± 3.41	0.011
Lifestyle							
	Smoking, n (%)	2423 (47.2)	174 (38.8)	422 (38.5)	475 (49.6)	1352 (51.3)	<0.001
	Drinking, n (%)	1198 (23.3)	102 (22.8)	236 (21.6)	231 (24.1)	629 (23.9)	0.429
Clinical factors							
	Hypertension, n (%)	3504 (68.2)	324 (72.3)	749 (68.4)	683 (71.4)	1748 (66.3)	0.006
	Diabetes mellitus, n (%)	1612 (31.4)	183 (40.8)	416 (38.0)	285 (29.8)	728 (27.6)	<0.001
Biochemical markers							
	Glucose, mmol/L (SD)	7.09 ± 3.12	7.24 ± 3.13	7.33 ± 3.29	6.83 ± 2.86	7.06 ± 3.12	0.003
	HCY, µmol/L [IQR]	17.98 [14.24–24.28]	12.70 [11.36–13.74]	12.80 [11.33–13.90]	20.82 [17.38–26.99]	21.12 [17.64–29.12]	<0.001
	LDL-C, mmol/L (SD)	2.37 ± 0.86	1.43 ± 0.27	2.69 ± 0.74	1.45 ± 0.25	2.72 ± 0.74	<0.001
	HDL-C, mmol/L (SD)	0.96 ± 0.26	0.97 ± 0.27	1.02 ± 0.27	0.91 ± 0.27	0.96 ± 0.24	<0.001
	TC, mmol/L (SD)	3.96 ± 1.09	2.97 ± 0.52	4.36 ± 1.02	2.95 ± 0.52	4.33 ± 1.01	<0.001
	TG, mmol/L (SD)	1.75 ± 1.20	1.61 ± 1.16	1.85 ± 1.39	1.54 ± 1.11	1.82 ± 1.15	<0.001
	Creatinine, µmol/L (SD)	74.22 ± 42.53	64.69 ± 18.86	64.39 ± 30.65	81.19 ± 55.84	77.38 ± 43.12	<0.001
Procedure							
	PCI, n (%)	3134 (61.0)	259 (57.8)	665 (60.7)	563 (58.8)	1647 (62.5)	0.102

Notes: Data are presented as mean (standard deviation, SD), median [interquartile range], or number (percentage). LDL-C, low-density lipoprotein cholesterol; HCY, homocysteine; BMI, body mass index; TC, total cholesterol; TG, triglycerides; PCI, percutaneous coronary intervention; HDL-C, high-density lipoprotein.

### 3.2 Association Between LDL-C and MACEs

**Fig. 2.**
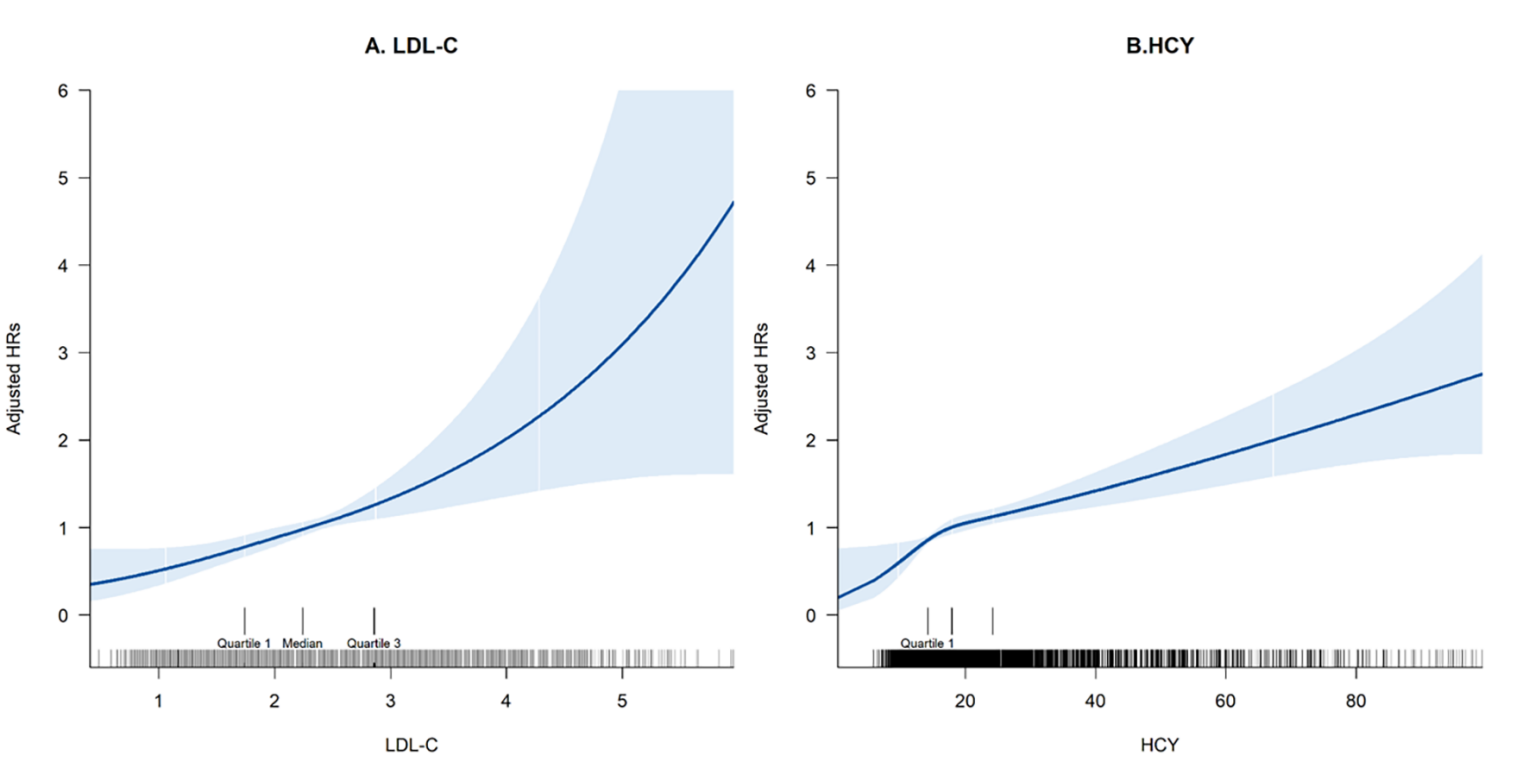
**Multivariable adjusted spline curves for the associations of (A) LDL-C and (B) HCY with the risk of MACEs**. HR, hazard ratio; MACEs, major adverse cardiovascular events.

Multivariable-adjusted spline curves indicated a positive continuous relationship between LDL-C concentration and the risk of MACEs (Fig. 2A). In the unadjusted model, participants with elevated LDL-C (≥1.8 mmol/L) had a 32% higher risk of MACEs relative to those with lower LDL-C (<1.8 mmol/L) (HR = 1.32, 95% CI: 1.12–1.57, *p* = 0.001). This relationship persisted after multivariable adjustment (adjusted HR = 1.38, 95% CI: 1.09–1.73, *p* = 0.006; Table 2).

**Table 2.** **Hazard ratios (HRs) for clinical outcomes by LDL-C and HCY categories**.

Group		n	Events, n (%)	Crude HR (95% CI)	*p* value	Adjusted HR†† (95% CI)	*p* value
LDL-C categories							
	LDL-C <1.8 mmol/L	1405	177 (12.6)	Ref	—	Ref	—
	LDL-C ≥1.8 mmol/L	3732	567 (15.2)	1.32 (1.12–1.57)	0.001	1.38 (1.09–1.73)	0.006
HCY categories							
	HCY <15 µmol/L	1543	139 (9.0)	Ref	—	Ref	—
	HCY ≥15 µmol/L	3594	605 (16.8)	1.56 (1.29–1.88)	<0.001	1.47 (1.19–1.81)	<0.001
Combined LDL-C and HCY							
	Low LDL-C & low HCY	448	37 (8.3)	Ref	—	Ref	—
	High LDL-C & low HCY	1095	102 (9.3)	1.23 (0.84–1.79)	0.285	1.35 (0.88–2.06)	0.169
	Low LDL-C & high HCY	957	140 (14.6)	1.46 (1.01–2.09)	0.043	1.44 (0.97–2.14)	0.067
	High LDL-C & high HCY	2637	465 (17.6)	1.93 (1.38–2.70)	<0.001	1.97 (1.34–2.90)	0.001
*p* for trend		—	—	—	<0.001	—	<0.001

Notes: ††Adjusted for age, sex, hypertension, diabetes mellitus, smoking, drinking, TC, TG, BMI and creatinine. CI, confidence interval; Ref, reference group.

### 3.3 Association Between HCY and MACEs

Fig. 2B illustrates the association between HCY levels and MACEs. Participants with high HCY (≥15 µmol/L) exhibited a significantly increased risk compared to those with low HCY (<15 µmol/L). In the unadjusted model, high HCY was associated with a 56% elevation in risk (HR = 1.56, 95% CI: 1.29–1.88, *p* < 0.001). Following adjustment, the association remained significant (adjusted HR = 1.47, 95% CI: 1.19–1.81, *p* < 0.001; Table 2).

### 3.4 The Combined Effect of LDL-C and HCY on MACEs

**Fig. 3.**
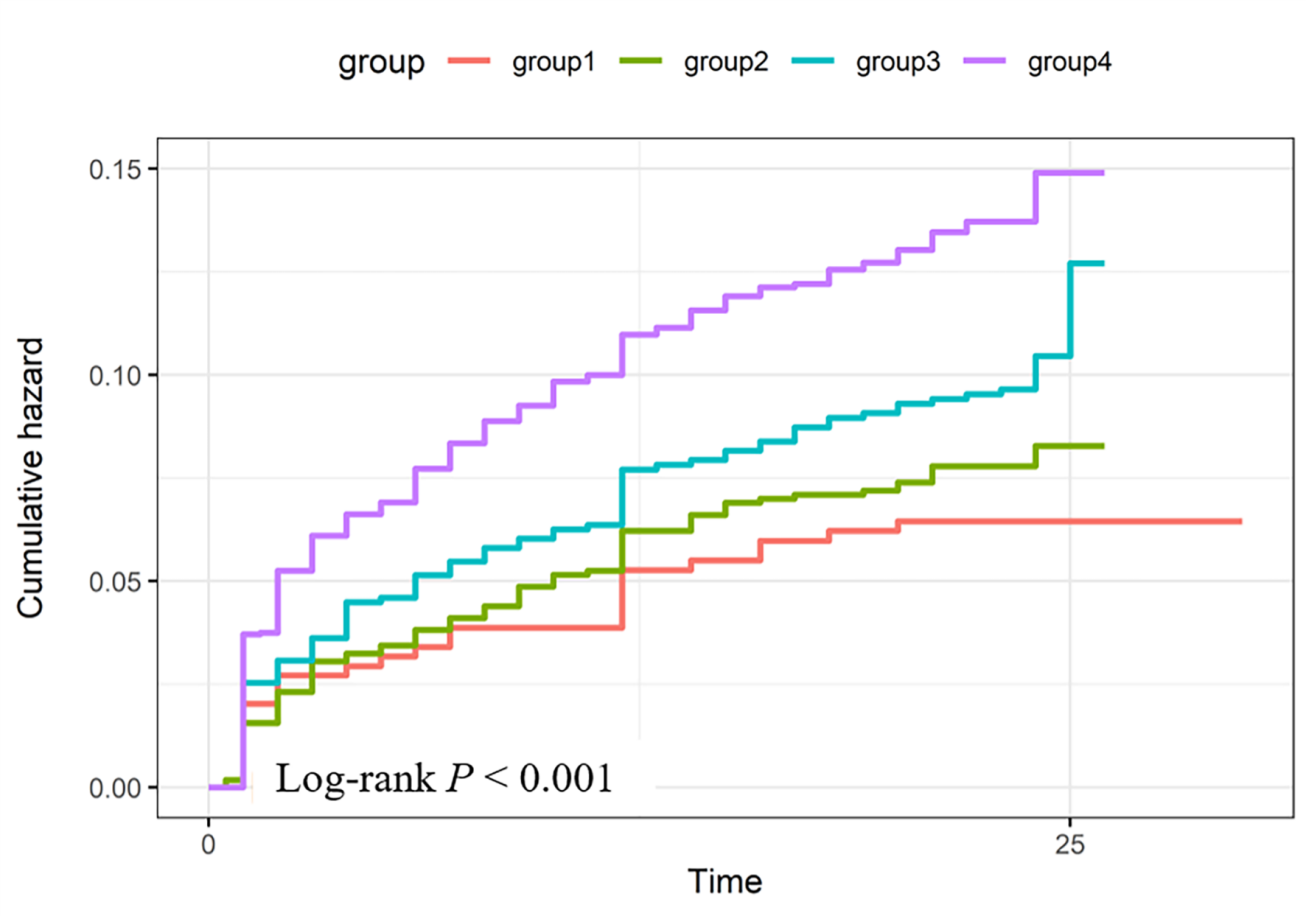
**Kaplan–Meier survival curves for cumulative incidence of MACEs stratified by LDL-C and HCY categories**. Groups include: Group1 (low LDL-C & low HCY), Group2 (high LDL-C & low HCY), Group3 (low LDL-C & high HCY), and Group4 (high LDL-C & high HCY). Log-rank test indicated significant differences among groups (*p* < 0.001).

Kaplan-Meier curves displayed a graded increase in cumulative MACE incidence across the groups (Fig. 3). The lowest risk was observed in the group with low LDL-C and low HCY, followed successively by high LDL-C & low HCY, low LDL-C & high HCY, and finally the high LDL-C & high HCY group (log-rank *p* < 0.001). In the adjusted Cox regression model, the group with both high LDL-C and high HCY had an almost twofold increased risk of MACEs compared to the reference group (adjusted HR = 1.97, 95% CI: 1.34–2.90, *p* = 0.001). The groups with high LDL-C & low HCY and low LDL-C & high HCY showed non-significant trends toward increased risk (adjusted HR = 1.35, 95% CI: 0.88–2.06, *p *= 0.169; and adjusted HR = 1.44, 95% CI: 0.97–2.14, *p* = 0.067, respectively; Table 2).

## 4. Discussion

This investigation confirms significant associations between LDL-C and HCY with the risk of MACEs in patients diagnosed with CHD. These observations are consistent with prior studies: LDL-C is a validated risk factor for atherosclerosis [2], and elevated HCY promotes endothelial dysfunction, oxidative stress, and thrombotic processes, thereby accelerating cardiovascular disease progression [8, 9, 10]. Importantly, our results further reveal a combined effect of LDL-C and HCY on MCAE risk. Patients with concurrent high LDL-C (≥1.8 mmol/L) and high HCY (≥15 µmol/L) demonstrated nearly twice the risk of MACEs compared to those with low levels of both. This finding emphasizes the potential clinical benefit of assessing both for improving risk stratification.

### 4.1 Relationship Between LDL-C and MACEs

LDL-C is universally acknowledged as a primary pathogenic agent in coronary atherosclerosis, a view substantiated by extensive evidence. For instance, investigations in renal transplant recipients have indicated that a high LDL-C/HDL-C ratio markedly increases cardiovascular morbidity and mortality, emphasizing its utility as a risk indicator [16]. Likewise, in individuals with high-risk hypercholesterolemia, LDL-C levels exhibit a linear correlation with cardiovascular disease incidence, affirming that reducing LDL-C decreases risk [17]. Furthermore, in patients presenting with acute coronary syndrome (ACS), reduced HDL-C levels are independently associated with increased cardiovascular event rates, underscoring the critical influence of lipid metabolism on clinical outcomes [18]. Despite effective LDL-C-lowering therapies, residual risk remains, implicating other factors such as triglycerides [19] and inflammation [20]. Our results reinforce LDL-C as a crucial predictor of cardiovascular risk in CHD patients and further identify elevated HCY as a factor contributing to residual risk.

### 4.2 Relationship Between HCY and MACEs

Elevated plasma HCY is associated with pro-atherogenic and pro-thrombotic mechanisms [21, 22]. A meta-analysis found that each 25% increase in plasma HCY concentration corresponds to a 10% greater risk of cardiovascular disease and a 20% increased risk of stroke [20]. Similarly, an increment of 5 µmol/L in HCY was associated with a 52% elevated risk of new-onset heart disease and a 32% higher mortality risk [23]. Clinically, high levels of HCY levels predict short-term adverse events in acute myocardial infarction patients [24] and are an independent predictor of MACEs in patients with ACS [25]. Moreover, the co-existence of hypertension and hyperhomocysteinemia has been strongly linked to carotid plaque development [26], amplifying overall cardiovascular risk. Although randomized controlled trials on HCY-lowering treatments have not uniformly shown clinical benefits [27], our findings suggest that elevated HCY retains clinical relevance.

### 4.3 Combined Effect of LDL-C and HCY on MACEs

The most compelling finding of this study was the combined effect of LDL-C and HCY on MACE risk. Patients with high concentrations of both indices the highest risk of MACEs. LDL-C primarily facilitates lipid accumulation within the arterial wall, advancing atherosclerosis and stenosis [28], whereas HCY aggravates vascular damage through endothelial dysfunction, oxidative stress, and inflammatory processes [26]. This combined effect stems from interconnected pathways where HCY-induced endothelial dysfunction and oxidative stress promote the retention and modification of LDL-C—a pivotal atherogenic step. Conversely, the inflammatory milieu from oxidized LDL exacerbates HCY-mediated metabolic disturbances, creating a vicious cycle that amplifies atherosclerosis.

The inconsistent outcomes of large-scale HCY-lowering trials with B vitamins [12], despite effectively reducing plasma HCY levels [29], may be explained by several factors: intervention timing may be too late in established CHD, HCY may be a marker of underlying pathology rather than a modifiable target, and benefits may be restricted to unselected genetic or nutritional subgroups. Our findings—that risk is greatest with concurrent hyperlipidemia—suggest future trials must account for this synergy. It is critical to emphasize that our results do not contradict the current clinical guidelines; they do not support the routine use of B-vitamin supplementation for CHD patients.

Current clinical guidelines prioritize LDL-C reduction as the foundation of secondary prevention [3, 4], yet residual risk remains substantial. Our study suggests that HCY assessment may help identify high-risk individuals. For patients with both high LDL-C and high HCY, aggressive lipid-lowering (e.g., with Proprotein Convertase Subtilisin/Kexin type 9 inhibitors) combined with HCY-lowering interventions might improve outcomes [30]. A dual-biomarker approach could enable more precise and personalized cardiovascular risk management.

Our findings advocate for a dual-biomarker strategy to identify high-risk CHD patients who may benefit from intensified management. However, optimizing secondary prevention extends beyond risk identification to ensuring long-term adherence to prescribed therapies. The challenge now lies in converting risk identification into sustained adherence. Digital strategies provide a solution: Digital Health Interventions, such as remote monitoring and messaging, boost medication adherence, as evidenced in post-ACS care [31]. Simultaneously, clinicians must steer patients toward trustworthy online health information [32]. Embedding these tools into care pathways is vital for ensuring long-term risk reduction.

## 5. Limitations

This study has several limitations. First, the retrospective, single-center design may affect the generalizability of the findings and introduce selection bias. Second, only baseline measurements of LDL-C and HCY were available; fluctuations over the follow-up period were not captured. Third, despite multivariate adjustment, residual confounding cannot be entirely ruled out due to unmeasured variables such as dietary habits, genetic factors, inflammatory markers, or medication compliance. Fourth, loss to follow-up may have introduced bias. Finally, the study cohort comprised only Chinese patients, potentially limiting the extrapolation of findings to other ethnic populations.

## 6. Conclusion

Both LDL-C and HCY were independent predictors of MACEs in CHD patients. When elevated together, they exhibited a combined effect, nearly doubling the risk of MACEs. These results support the clinical utility of combined biomarker assessment for improved cardiovascular risk stratification. Future prospective, multicenter studies are needed to validate these findings and explore integrated treatment strategies targeting both LDL-C and HCY.

## Availability of Data and Materials

The datasets generated during and analyzed during the current study are available from the corresponding author on reasonable request.

## References

[1] Martin SS, Aday AW, Almarzooq ZI, Anderson CAM, Arora P, Avery CL (2024). 2024 Heart Disease and Stroke Statistics: A Report of US and Global Data From the American Heart Association. *Circulation*.

[2] Ference BA, Ginsberg HN, Graham I, Ray KK, Packard CJ, Bruckert E (2017). Low-density lipoproteins cause atherosclerotic cardiovascular disease. 1. Evidence from genetic, epidemiologic, and clinical studies. A consensus statement from the European Atherosclerosis Society Consensus Panel. *European Heart Journal*.

[3] Grundy SM, Stone NJ, Bailey AL, Beam C, Birtcher KK, Blumenthal RS (2019). 2018 AHA/ACC/AACVPR/AAPA/ABC/ACPM/ADA/AGS/APhA/ASPC/NLA/PCNA Guideline on the Management of Blood Cholesterol: Executive Summary: A Report of the American College of Cardiology/American Heart Association Task Force on Clinical Practice Guidelines. *Journal of the American College of Cardiology*.

[4] Visseren FLJ, Mach F, Smulders YM, Carballo D, Koskinas KC, Bäck M (2021). 2021 ESC Guidelines on cardiovascular disease prevention in clinical practice. *European Heart Journal*.

[5] Peng YP, Huang MY, Xue YJ, Pan JL, Lin C (2020). Association of Hyperhomocysteinemia with Increased Coronary Microcirculatory Resistance and Poor Short-Term Prognosis of Patients with Acute Myocardial Infarction after Elective Percutaneous Coronary Intervention. *BioMed Research International*.

[6] Agoston-Coldea L, Mocan T, Gatfosse M, Lupu S, Dumitrascu DL (2011). Plasma homocysteine and the severity of heart failure in patients with previous myocardial infarction. *Cardiology Journal*.

[7] He Y, Li Y, Chen Y, Feng L, Nie Z (2014). Homocysteine level and risk of different stroke types: a meta-analysis of prospective observational studies. *Nutrition, Metabolism, and Cardiovascular Diseases: NMCD*.

[8] He L, Zeng H, Li F, Feng J, Liu S, Liu J (2010). Homocysteine impairs coronary artery endothelial function by inhibiting tetrahydrobiopterin in patients with hyperhomocysteinemia. *American Journal of Physiology. Endocrinology and Metabolism*.

[9] Guo G, Sun W, Liu G, Zheng H, Zhao J (2018). Comparison of oxidative stress biomarkers in hypertensive patients with or without hyperhomocysteinemia. *Clinical and Experimental Hypertension (New York, NY: 1993)*.

[10] Xie R, Jia D, Gao C, Zhou J, Sui H, Wei X (2014). Homocysteine induces procoagulant activity of red blood cells via phosphatidylserine exposure and microparticles generation. *Amino Acids*.

[11] Sun Y, Chien KL, Hsu HC, Su TC, Chen MF, Lee YT (2009). Use of serum homocysteine to predict stroke, coronary heart disease and death in ethnic Chinese. 12-year prospective cohort study. *Circulation Journal: Official Journal of the Japanese Circulation Society*.

[12] Herrmann W, Herrmann M (2022). The Controversial Role of HCY and Vitamin B Deficiency in Cardiovascular Diseases. *Nutrients*.

[13] Apostolov EO, Ok E, Burns S, Nawaz S, Savenka A, Shah SV (2013). Carbamylated-oxidized LDL: proatherosclerotic effects on endothelial cells and macrophages. *Journal of Atherosclerosis and Thrombosis*.

[14] Wang X, Ma X, Zeng Y, Xu L, Zhang M (2023). Hypermethylation of the CTRP9 promoter region promotes Hcy induced VSMC lipid deposition and foam cell formation via negatively regulating ER stress. *Scientific Reports*.

[15] Yao Y, Liu F, Wang Y, Liu Z (2020). Lipid levels and risk of new-onset atrial fibrillation: A systematic review and dose-response meta-analysis. *Clinical Cardiology*.

[16] Nagel N, Rahamimov R, Bielopolski D, Steinmetz T, Skalsky K, Zingerman B (2024). Analysis of the Correlation between Hypercholesterolemia and Increased Cardiovascular Morbidity and Mortality among Adult Kidney Transplant Recipients. *Kidney & Blood Pressure Research*.

[17] Daida H, Teramoto T, Kitagawa Y, Matsushita Y, Sugihara M (2014). The relationship between low-density lipoprotein cholesterol levels and the incidence of cardiovascular disease in high-risk patients treated with pravastatin: main results of the APPROACH-J study. *International Heart Journal*.

[18] Nakazawa M, Arashi H, Yamaguchi J, Ogawa H, Hagiwara N (2020). Lower levels of high-density lipoprotein cholesterol are associated with increased cardiovascular events in patients with acute coronary syndrome. *Atherosclerosis*.

[19] Nichols GA, Philip S, Reynolds K, Granowitz CB, Fazio S (2019). Increased residual cardiovascular risk in patients with diabetes and high versus normal triglycerides despite statin-controlled LDL cholesterol. *Diabetes, Obesity & Metabolism*.

[20] Di Muro FM, Vogel B, Sartori S, Bay B, Oliva A, Feng Y (2025). Prognostic impact of residual inflammatory and triglyceride risk in statin-treated patients with well-controlled LDL cholesterol and atherosclerotic cardiovascular disease. *European Journal of Preventive Cardiology*.

[21] Al-Obaidi MK, Philippou H, Stubbs PJ, Adami A, Amersey R, Noble MM (2000). Relationships between homocysteine, factor VIIa, and thrombin generation in acute coronary syndromes. *Circulation*.

[22] Hajjar KA (1993). Homocysteine-induced modulation of tissue plasminogen activator binding to its endothelial cell membrane receptor. *The Journal of Clinical Investigation*.

[23] Ostrakhovitch EA, Tabibzadeh S (2019). Homocysteine and age-associated disorders. *Ageing Research Reviews*.

[24] Ma Y, Li L, Geng XB, Hong Y, Shang XM, Tan Z (2016). Correlation Between Hyperhomocysteinemia and Outcomes of Patients With Acute Myocardial Infarction. *American Journal of Therapeutics*.

[25] Wei M, Wang L, Liu YS, Zheng MQ, Ma FF, Qi YC (2019). Homocysteine as a potential predictive factor for high major adverse cardiovascular events risk in female patients with premature acute coronary syndrome. *Medicine*.

[26] Chen Z, Wang F, Zheng Y, Zeng Q, Liu H (2016). H-type hypertension is an important risk factor of carotid atherosclerotic plaques. *Clinical and Experimental Hypertension (New York, NY: 1993)*.

[27] Lonn E, Yusuf S, Arnold MJ, Sheridan P, Pogue J, Micks M (2006). Homocysteine lowering with folic acid and B vitamins in vascular disease. *The New England Journal of Medicine*.

[28] Bianconi V, Banach M, Pirro M, International Lipid Expert Panel (ILEP) (2021). Bianconi V, Banach M, Pirro M, International Lipid Expert Panel (ILEP). Why patients with familial hypercholesterolemia are at high cardiovascular risk? Beyond LDL-C levels. *Trends in Cardiovascular Medicine*.

[29] Li M, Ren R, Wang K, Wang S, Chow A, Yang AK (2025). Effects of B Vitamins on Homocysteine Lowering and Thrombotic Risk Reduction-A Review of Randomized Controlled Trials Published Since January 1996. *Nutrients*.

[30] Sabatine MS, Giugliano RP, Keech AC, Honarpour N, Wiviott SD, Murphy SA (2017). Evolocumab and Clinical Outcomes in Patients with Cardiovascular Disease. *The New England Journal of Medicine*.

[31] Şaylık F, Çınar T, Hayıroğlu Mİ, Tekkeşin Aİ (2023). Digital Health Interventions in Patient Management Following Acute Coronary Syndrome: A Meta-Analysis of the Literature. *Anatolian Journal of Cardiology*.

[32] Hayıroğlu Mİ, Çinier G, Keser N, Uzun M, Karagoz A, Fak AS (2020). Evaluation of websites reached using Google in the modern digital era related to approach to cholesterol. *Turk Kardiyoloji Dernegi Arsivi: Turk Kardiyoloji Derneginin Yayin Organidir*.

